# Outcomes of Hallux Valgus Corrective Osteotomy: A Tertiary Hospital Experience

**DOI:** 10.7759/cureus.46422

**Published:** 2023-10-03

**Authors:** Ali S Alshehri, Faisal A Alzahrani, Ziad A Aljaafri, Nada A Shalash

**Affiliations:** 1 Orthopedic Surgery, King Abdulaziz Medical City, Ministry of National Guard – Health Affairs, Riyadh, SAU; 2 Orthopedic Surgery, King Abdullah International Medical Research Center, Riyadh, SAU; 3 College of Medicine, King Saud Bin Abdulaziz University for Health Sciences, Riyadh, SAU

**Keywords:** outcome studies, surgical outcome, hallux valgus surgery, corrective osteotomy, hallux valgus

## Abstract

Background: A common form of forefoot deformity, hallux valgus (HV) is characterized by a prominent first metatarsal head, lateral deviation of the hallux, and medial deviation of the first metatarsal bone. In the case of HV, corrective osteotomies are performed with good results and patient satisfaction.

Methods: A retrospective cohort study of patients who underwent corrective osteotomy for hallux valgus from 2016 to 2022 was conducted at King Abdulaziz Medical City (KAMC), Riyadh, Saudi Arabia. Data were collected by chart review using the BestCARE system. IBM SPSS Statistics for Windows, Version 23.0 (Released 2015; IBM Corp., Armonk, New York, United States) was used for statistical analysis.

Results: Our study included 166 patients. The mean age of the patients was found to be 41.3 years old and about 152 (91.6%) of them were females. The most frequently reported comorbidity was hypertension (10.2%). The mean hallux valgus angle was found to be 36.1 ± 9.9 and the mean intermetatarsal angle was found to be 15 ± 4.4 degrees. Seventy-six (45.8%) patients underwent nonoperative management first. The mean age at diagnosis among males was found to be 28.5 ± 11.3 years and among females was 37.9 ± 14.4 years; a significant difference between means was noted (p-value = 0.019) with mean age at diagnosis in males being significantly lesser than in females.

Conclusion: Significant improvement and reduction were seen in HV angle post surgery. Nearly half of the patients underwent nonoperative management first. Age at diagnosis is significantly younger in males compared to females.

## Introduction

Hallux valgus (HV) is a common form of forefoot deformity that usually affects women more than men [1,2]. Usually, predisposing factors play a role in having the condition such as gastrocnemius equinus, abnormal foot mechanics, joint hypermobility, short first metatarsal, dorsiflexed first metatarsal, flexible or rigid forefoot varus, rigid or flexible pes planovalgus, and genetics [1,3]. However, the exact etiology remains uncertain.

HV epidemiology has been reported in multiple international studies, with a range exceeding 25% [4-6]. There is a female predominance. One of the meta-analyses conducted showed a higher prevalence of HV among the elderly compared to other age groups [7,8]. A local study reported the prevalence of HV in the central region of Saudi Arabia to be around 43% with higher female prevalence [9].

Medial deviation of the first metatarsal bone, lateral deviation of the hallux, and a prominent first metatarsal head are the main features of this condition. Instability and insufficiency from the distal phalanx to the talonavicular joint, or anywhere along the first ray, can result in HV [10,11]. HV is usually a clinical diagnosis. Furthermore, no laboratory tests are routinely required to be done, unless an underlying issue is suspected, for instance, systemic or metabolic disorders. However, radiological assessment is necessary to evaluate the severity as well for surgical planning, and it is the gold standard for diagnosing HV [12]. Because it has been demonstrated that the HV angle (HVA) and the intermetatarsal angle (IMA) are best able to correlate with the magnitude of the deformity and assess the severity of HV, they are the parameters that are utilized the most frequently in clinical practice [12], in comparison to distal metatarsal articular angle (DMAA), which has been considered as less reliable in multiple studies [3,13,14].

Mild HV can be considered if HVA < 20° and the IMA is between 9-11° in which distal osteotomy can be done with or without soft tissue involvement. For moderate HV, HVA is 20-40°, and the IMA is between 11-16° where proximal osteotomy is considered with or without soft tissue being involved. Finally, for severe HV, HVA is > 40° and the IMA is > 16° therefore, proximal osteotomy or first tarsometatarsal arthrodesis is done with or without the involvement of soft tissue [3,15,16]. 

The weight-bearing anteroposterior (AP), lateral oblique, lateral, and sesamoid axial views are typically used to classify the severity of HV. The AP view is especially considered for measuring the HVA and IMA, in which normally the values are <15° and <9°, respectively. Also, the normal value of DMAA should be kept in consideration, which is <10°. Furthermore, lateral obliques are utilized to assess the uniformity, trabeculation, and density of the bone, lateral view is mainly used to evaluate the first meta-tarsal position and dorsal presence of osteophytes/exotosis if any is found, and sesamoid axial projection for subluxation of the sesamoid or any degenerative changes affecting the cristae [1,3,15,17].

Corrective osteotomy is done for HV with great outcomes and patient satisfaction. However, few studies have shown the outcome of the cases. Therefore, we would like to study the outcome of corrective surgeries done for HV at our institution, King Abdulaziz Medical City (KAMC), Riyadh, Saudi Arabia.

## Materials and methods

A retrospective cohort study was conducted at KAMC, a tertiary hospital in Riyadh, Saudi Arabia. The sample size was determined by including all patients who underwent corrective osteotomy for HV from 2016 to 2022. No exclusion criteria were implemented. 

Data were collected through the BestCARE system in KAMC. We used a data collection sheet that was prepared by the research team based on data of interest. The data collection sheet included demographics, comorbidities, age at diagnosis, preoperative and postoperative HVA and IMA, modality of conservative management if started, complications, and recurrence if found. 

All the data were collected through Microsoft Excel (Microsoft Corporation, Redmond, Washington, United States) and transferred for analysis. Data were checked for any missing information and new variables were recorded and computed based on the data extracted. Statistical analysis was performed using IBM SPSS Statistics for Windows, Version 25.0 (Released 2017; IBM Corp., Armonk, New York, United States). Frequencies and percentages were used to detail categorical variables, whereas continuous variables were examined by the mean and standard deviation. A p-value <0.05 was considered to report the statistical significance.

This study was approved by the King Abdullah International Medical Research Center Institutional Review Board (approval number IRB/0894/23). The requirement for informed consent was waived due to the retrospective nature of this study. No identifying data were asked, ensuring privacy and confidentiality. All data were kept safe, with only the authors having access to the research data.

## Results

Our study included 166 patients. The mean age of the patients was found to be 41.3 ± 14.9 years (range, 15-78 years old). The mean BMI of the patients was found to be 27.3 ± 6.12 Kg/m^2^. A total of 152 (91.6%) patients were females and 14 (8.4%) were males. The most frequently reported comorbidity was found to be hypertension, which was found in 17 (10.2%) patients followed by hypothyroidism in 12 (7.2%), diabetes mellitus in 11 (6.6%), rheumatoid arthritis in five (3%), steroid use in three (1.8%), stroke in two (1.2%), and peripheral vascular disease (PVD) in one (0.6%) patients; 52 (31.3%) patients were found to be having other co-morbidities (Table 1).

**Table 1:** Demographic data of the study patients (n=166) Data has been represented as numbers (N) and percentages (%), and mean ± SD where mentioned

Variable	Overall
Age, mean ± SD (Range)	41.3 ± 14.9 years (15 – 78)
BMI, mean ± SD	27.3 ± 6.12 kg/m^2^
Gender, n (%)	
Male	14 (8.4)
Female	152 (91.6)
Patient risks factors, n (%)	
Diabetes	11 (6.6)
Hypertension	17 (10.2)
Hypothyroidism	12 (7.2)
Rheumatoid arthritis	5 (3)
Stroke	2 (1.2)
Peripheral vascular disease (PVD)	1 (0.6)
Steroid use	3 (1.8)
Other comorbidities	52 (31.3)

**Figure 1:**
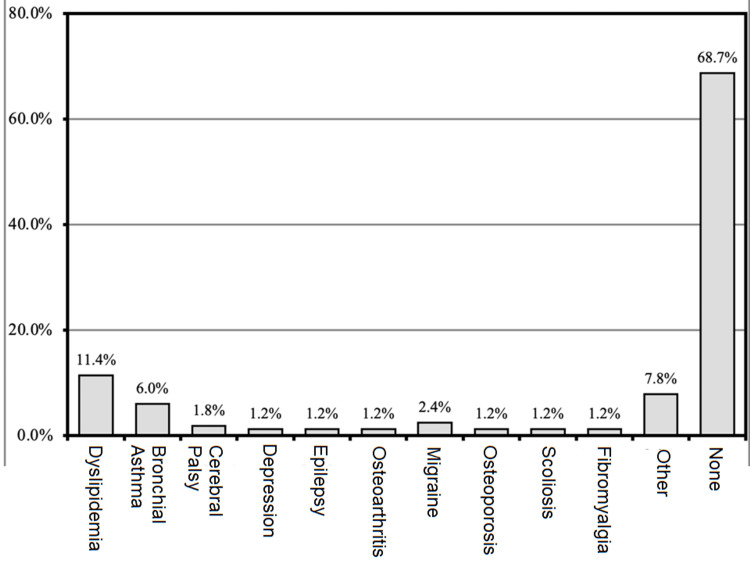
Other comorbidities (n=166)

Other co-morbidities include dyslipidemia in 19 (11.4%) of the patients. Depression, epilepsy, osteoarthritis, osteoporosis, and scoliosis in two (1.2%) patients each, four (2.4%) were found to be having migraine, 13 (7.8%) had other co-morbidities, and 114 (68.7%) had none (Figure 1).

The mean age at diagnosis with HV deformity was found to be 37.1 ± 14.4 years. A total of 162 (97.6%) patients had acquired HV deformity whereas four (2.4%) had congenital HV deformity. HV deformity was bilateral in 100 (60%) patients and was unilateral in 66 (39.8%). The mean HVA was found to be 36.1 ± 9.9 and the mean IMA was found to be 15 ± 4.4 degrees. Seventy-six (45.8%) patients underwent nonoperative management first before undergoing corrective osteotomy. Nonoperative management included physiotherapy (PT) and shoe modifications, used by 56 (33.7%) patients, insoles and PT used by 14 (8.4%) patients, and silicone splints and toe spacers utilized by three (1.8%) patients. The average HVA post operation was found to be 15.6 ± 7.1 degrees. The mean IMA post operation was found to be 6.9 ± 2.8 degrees. A total of 27 (16.3%) had complications and the rest 139 (83.7%) were with no complications. Twenty-three (13.9%) patients suffered from recurrence and the rest 143 (86.1%) had no recurrence (Table 2).

**Table 2:** Corrective osteotomy surgery information, complications, and recurrence HVA: hallux valgus angle; IMA: intermetatarsal angle; PT: physiotherapy Data has been represented as numbers (N) and percentages (%), and mean ± SD where mentioned

Variable	N (%)
Age at diagnosis (years), mean ± SD	37.1 ± 14.4
Acquired / Congenital, n (%)	
Acquired	162 (97.6)
Congenital	4 (2.4)
Unilateral / Bilateral, n (%)	
Unilateral	66 (39.8)
Bilateral	100 (60.2)
Hallux valgus angle (HVA), mean ± SD	36.1 ± 9.9
Intermetatarsal angle (IMA), mean ± SD	15.3 ± 4.4
Nonoperative management, n (%)	
Yes	76 (45.8)
No	90 (54.2)
Method, n (%)	
PT & Shoe modification	56 (33.7)
Insoles & PT	14 (8.4)
Silicone splint	3 (1.8)
Toe spacer	3 (1.8)
NA	90 (54.2)
HVA post-surgery, mean ± SD	15.6 ± 7.1
IMA post-surgery, mean ± SD	6. 9 ± 2.8
Complications, n (%)	
Yes	27 (16.3)
No	139 (83.7)
Recurrence, n (%)	
Yes	23 (13.9)
No	143 (86.1)

In regard to complications of HV surgery, pain was reported by six (3.6%) patients, arthritis and stiffness were reported by four (2.4%) patients each, and metatarsalgia was reported in three (1.8%) patients. Neuromas, surgical site infection (SSI), and subluxation were found in one (0.6%) patient each. Most of the patients were with no complications (83.7%).

Using an independent sample t-test for comparison between the mean age at diagnosis for both genders, the mean age at diagnosis among males was found to be 28.5 ± 11.3 years and the mean age at diagnosis among females was 37.9 ± 14.4 years; a significant difference between means was noted (p-value= 0.019) with mean age at diagnosis being significantly lower in males than in females. The mean age at diagnosis in those who were less than or equal to 40 years old was 25.5 ± 6.5 and the mean age for those who were more than 40 years old was 49.8 ± 8.8. There was a significant difference in age at diagnosis, which was earlier in those who were less than 40 years old. Gender and age group were not significantly associated with the cause of HV deformity (p-value= 0.299 and 0.122, respectively).

No significant differences were noted between gender and age group in the laterality of the deformity (p-value= 0.804 and 0.076). There was no difference in means of HVA among both genders and age groups (p-value= 0.454 and 0.739). Gender and age were not significantly associated with conservative management (p-value= 0.056 and 0.499, respectively).

No significant mean differences were noted in HVA in both gender and age (p-value= 0.692 and 0.739, respectively). Complications were not found to be associated with specific gender or age (p-value=0.471 and 0.080, respectively). There was no difference in the recurrence of HV regarding groups of gender and age (p-value= 1.000 and 0.355, respectively) (Table 3).

**Table 3:** Comparison of the results based on gender and different age groups ^*^ p-values calculated using Fisher's exact test (F), independent samples t test (t), and chi square test HVA: hallux valgus angle; IMA: intermetatarsal angle; PT: physiotherapy Data has been represented as numbers (N) and percentages (%), and mean ± SD where mentioned

Variable	Gender		p-value	Age group		p-value
	Male (n=14)	Female (n=152)		≤ 40 years (n=87)	> 40 years (n=79)	
Age at diagnosis (years), mean ± SD	28.5 ± 11.3	37.9 ± 14.4	0.019^t^	25.5 ± 6.5	49.8 ± 8.8	< 0.001^t^
Acquired/Congenital, n (%)						
Acquired	13 (92.9)	149 (98)	0.299^F^	83 (95.4)	79 (100)	0.122^F^
Congenital	1 (7.1)	3 (2)		4 (4.6)	0 (0)	
Unilateral/Bilateral, n (%)						
Unilateral	6 (42.9)	60 (39.5)	0.804	29 (33.3)	37 (46.8)	0.076
Bilateral	8 (57.1)	92 (60.5)		58 (66.7)	42 (53.2)	
Hallux valgus angle (HVA), mean ± SD	35.0 ± 9.7	36.2 ± 9.9	0.692^t^	36.3 ± 10.7	35.8 ± 9.0	0.739^t^
Intermetatarsal angle (IMA), mean ± SD	14.3 ± 4.0	15.3 ± 4.4	0.454^t^	14.9 ± 4.3	15.7 ± 4.5	0.228^t^
Conservative management, n (%)						
Yes	3 (21.4)	73 (48)	0.056	42 (48.3)	34 (43)	0.499
No	11 (78.6)	79 (52)		45 (51.7)	45 (57)	
Method, n (%)						
PT & shoe modification	3 (100)	53 (72.6)	0.693^F^	30 (71.4)	26 (76.5)	0.768^F^
Insoles & PT	0 (0)	14 (19.2)		9 (21.4)	5 (14.7)	
Silicone splint	0 (0)	3 (4.1)		1 (2.4)	2 (5.9)	
Toe spacer	0 (0)	3 (4.1)		2 (4.8)	1 (2.9)	
HVA post-surgery, mean ± SD	15.8 ± 8.7	15.5 ± 7.0	0.911^t^	15.3 ± 7.6	15.9 ± 6.6	0.622^t^
IMA post-surgery, mean ± SD	7.6 ± 3.3	6.9 ± 2.7	0.363^t^	6.4 ± 2.3	7.5 ± 3.2	0.020^t^
Complications, n (%)						
Yes	1 (7.1)	26 (17.1)	0.471^F^	10 (11.5)	17 (21.5)	0.080
No	13 (92.9)	126 (82.9)		77 (88.5)	62 (78.5)	
Recurrence, n (%)						
Yes	2 (14.3)	21 (13.8)	1.000^F^	10 (11.5)	13 (16.5)	0.355
No	12 (85.7)	131 (86.2)		77 (88.5)	66 (83.5)	

## Discussion

Studying outcomes of HV corrective surgery is important as it shows the benefits of surgery, its effects on quality of life, patient's preferences after various treatment modalities, and their response to certain treatments [18]. Additionally, it aids in identifying the frequency of post-surgical complications and reduces factors that lead to the occurrence of complications [18].

The mean age of the patients was found to be 41.3 years old. The vast majority (91.6%) of the patients were females and the rest were males. The most frequently reported comorbidity was found to be hypertension, which was found in 10.2% of the patients followed by hypothyroidism in 7.2% of the patients, diabetes mellitus in 6.6%, and rheumatoid arthritis in five (3%) patients. Autoimmune disorders such as rheumatoid arthritis were also noted in the study conducted by Louwerens et al., which reported the link between rheumatoid arthritis and HV deformity [19].

The mean age at diagnosis of HV deformity was found to be 37.1 years and this was found to be consistent with the findings reported in the study carried out by Coughlin et al., in which the mean age at diagnosis was between the second and the fifth decade of age [3]. In a study by Piqué-Vidal and Vila, HV deformity was found to be acquired in about 97.6% of patients, whereas in 2.4% of patients, it was congenital. HV deformity was bilateral in less than two-thirds (60%) of the patients and it was unilateral in more than one-third (39.8%). The mean HVA was found to be 36.1° and this was regarded as severe in the study [20]. Less than half (45.8%) of the patients underwent nonoperative management. The type of nonoperative management was physiotherapy (PT) and shoe modifications, which were utilized by about one-third (33.7%) of patients, insoles and PT in 8.4% of the patients, silicone splints and toe spacers were used by 1.8% of the patients each, and nonoperative treatment was recommended to 92% of the patients in the parallel study by Hurn et al. [21]. The mean HVA post operation was found to be 15.6°. Twenty-seven (16.3%) patients had complications. About 13.9% of the patients suffered from recurrence and this was found to be consistent with the findings reported in the congruent study by Park et al., in which 17.1% of the patients had recurrence [22].

Regarding complications of HV surgery, hallux arthritis, and stiffness were reported by 2.4% of the patients each, metatarsalgia was reported in 1.8% of the patients, and similar findings were reported in the study carried out by Miranda et al. in which 18.47% of the patients suffered from recurrence [23].

No significant mean differences were noted in HVA regarding groups of both gender and age. Complications were not found to be associated with specific gender or age. No difference in the recurrence of HV regarding groups of gender and age. The mean age at diagnosis in gender groups was found to be significant, with a significantly younger mean age at diagnosis in males compared to females, and this was found to be contradictory to the findings mentioned in the study conducted by Dunn et al., in which most HV deformity was mostly diagnosed in young females [6], and these differences could be attributed to differences in the studied sample.

Regarding the limitations faced in this study, the data were gathered from one center, theoretically limiting the generalizability of the findings. This study requires expansion in terms of sample size and the included centers, adding other factors to be studied as well for a precise assessment of the outcomes of corrective osteotomies for HV and also to assess the satisfaction rate of the patients who underwent the surgical intervention.

## Conclusions

Significant improvement and reduction was seen in HVA post surgery. Nearly half of the patients underwent nonoperative management first. Complications were reported to a lesser extent and the most common surgical complication noted was post-surgical pain followed by hallux arthritis and stiffness. Age at diagnosis is significantly younger in males compared to females.
